# The effective remediation of heavy metal-laden wastewater by employing cement dust derived from industrial activities as a sorbent

**DOI:** 10.1039/d5ra06620d

**Published:** 2025-11-10

**Authors:** Eslam Syala, Wagih A. Sadik, Abdel-Ghaffar M. El-Demerdash, Waffa Mekhamer, M. Essam El-Rafey

**Affiliations:** a Department of Materials Science, Institute of Graduate Studies and Researches (IGSR), Alexandria University 163 Horreya Avenue, Shatby 21526 Alexandria Egypt Eslam.Syala@alexu.edu.eg

## Abstract

As a continuation of our earlier study, “The effective treatment of dye-containing simulated wastewater by using the cement kiln dust as an industrial waste adsorbent”, the present work investigates the purification of wastewater containing heavy metals (HMs) using the industrial waste byproduct, cement kiln dust (CKD), on both laboratory and industrial scales. The adsorption of HMs was investigated through isothermal equilibrium batch experiments. This study primarily focused on detecting and removing traces of lead (Pb) and cadmium (Cd), with the removal process examined using X-ray fluorescence, scanning electron microscopy, Fourier-transform infrared spectroscopy, thermogravimetric analysis, and X-ray diffractometry techniques. The adsorption of both metals was examined under varying contact times, pH levels, temperatures, and several initial concentrations of Pb and Cd. The results revealed that maximum sorption was acquired after 80 minutes at pH 8 and 55 °C. Linear and nonlinear pseudo-first-order, pseudo-second-order, and intraparticle diffusion kinetic models were employed. The best fit of the kinetic data was achieved using the pseudo-second-order model, with a regression coefficient (*R*^2^) of 0.997. Analyzing the thermodynamic parameters revealed that the adsorption activity was endothermic and energetically favorable. The adsorption process was found to be the formation of a homogeneous monolayer/heterogeneous multilayer spread over the points of activation of the CKD grains, as detected from the analysis of the linear/nonlinear patterns of the Langmuir and Freundlich isotherms, with preference for the Freundlich model in describing the adsorption process based on its higher *R*^2^. The adsorption capacities were 403.7 and 362.54 mg g^−1^, respectively, for the Pb and Cd metals. A real industrial wastewater sample was treated with CKD to assess the removal process, and the HMs removal efficiency was found to be between 86.70 and 90.77%. CKD as an adsorbent has not been extensively studied before; however, all the findings revealed that it is a very low-cost adsorbent and that it is practical for the powerful elimination of HM contamination from real and/or simulated industrial wastewater.

## Abstract 2

This work studies the purification of wastewater containing heavy metals by cement dust as a continuation of the paper “The effective treatment of dye-containing simulated wastewater by using the cement kiln dust as an industrial waste adsorbent”.

## Graphical abstract

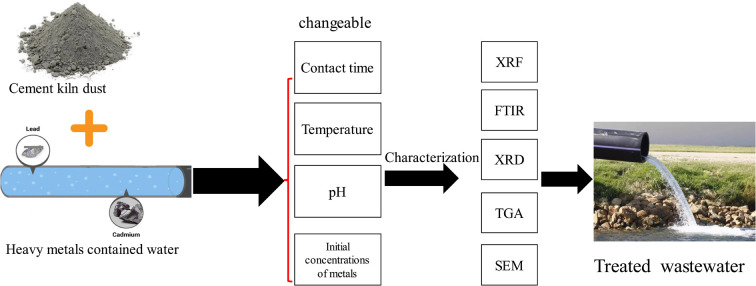

## 1 Introduction

The rapid spread and diversification of industrial activities has led to the emission of hazardous pollutants, which in turn have aggravated environmental and health problems. Among these industrial effluents, heavy metals (HMs) are considered to be pollutants that are toxic for human beings and ecosystems. Industrial and mining activities are major contributors to HM pollution of water bodies and sewage networks.^1^ These heavy metals contaminate the soil, air, and water, posing risks to human health and ecological stability when their concentrations exceed the permissible limits.^2^ In addition, HMs are characterised by their minute size, nanoscale dimensions, pronounced toxicity, resistance to biodegradation, and long-lasting environmental persistence.^3^ Thus, it is essential to decontaminate sources of HMs (especially HMs-containing wastewater) to reduce both the effort and costs required for treatment and to mitigate the subsequent effects of contamination.^4^ Both lead and cadmium are common toxic HMs that humans and other beings can be exposed to through food, drinking water, ambient air, industrial materials, and consumable products. Prolonged exposure to both metals may adversely impact the health of humans and other beings. Cadmium and lead and their compounds are classed as group 1 carcinogens and probable human carcinogens (group 2A), respectively, in line with the guidelines from the International Agency for Research on Cancer (IACR).^1,5,6^ Recycling (which is considered difficult and not economically rewarding), chemical precipitation, ultrafiltration, coagulation and flocculation, anaerobic and aerobic digestion, extraction, catalytic degradation, ion exchange, reverse osmosis, membrane separation, and adsorption are the most popular treatment techniques for the removal of HMs.^7,8^ Ion exchange, either natural or artificial, is among the most applied removal mechanisms and is widespread in the field. Several materials can be employed in this regard.^4^ Cement kiln dust (CKD) is a cement manufacturing side-product solid waste that is generated from rotary kiln furnaces and is captured by electrical filters. It is generated in large amounts, up to about 7–10% of cement production.^9,10^ Most of these amounts are damped and deposited in landfill sites, which presents a burden on the environment. CKD can be applied as an absorber for HM cations.^1,4,11–14^ Rather than for HMs removal, CKD can also be applied for the capture of radioactive uranium from aqueous solution^15^ as well as rare earth metals.^16^ Previous attempts to use CKD for the removal of a single heavy metal required prior treatment of the CKD surface to enhance the removal process,^17^ which increased both effort and cost. The present research reported here applied CKD as it is, without any treatment, for the removal of more than one heavy metal, which requires less effort and has economic advantages. The CKD was characterized by utilizing varied techniques such as XRF, XRD, FTIR, and SEM. The elimination of lead and cadmium from simulated (experimental) wastewater was examined under varying conditions, including different masses of CKD (g), temperatures (°C), contact times (min), pH levels, and several initial concentrations of heavy metals (ppm). Each factor of the aforementioned parameters was studied while simultaneously keeping the others unchanged. The mode of removal was explored through batch experiments. Pseudo-first/pseudo-second-order, as well as intraparticle diffusion kinetic models, were applied and studied. Langmuir and Freundlich adsorption models were applied to detect the HMs adsorption on the outside of the adsorbent (CKD). Thermodynamic parameters were also examined to assess the HMs removal. The present study complements a previous article (the effective treatment of dye-containing simulated wastewater by using the cement kiln dust as an industrial waste adsorbent) by attempting to conduct a comprehensive environmental and economic study on the use of cement kiln dust.

## 2 Experimental approaches (methodology)

### 2.1 The starting materials

The needed amount of CKD for the experiments was bought from the Alexandria Portland Cement Company (TITAN), in the west of Alexandria province, Egypt. The required amount of CKD for the experiments was obtained from the Alexandria Portland Cement Company (TITAN), located west of Alexandria, Egypt. Lead nitrate and Cadmium nitrate tetrahydrate crystalline powder, the sources of lead and cadmium metals, with purity ≥99.0% were ordered from Fisher Scientific, while sodium hydroxide (NaOH) pellets, potassium nitrate (KNO_3_), nitric acid (HNO_3_), and hydrochloric acid (HCl) were all brought from E. Merck.

### 2.2 Methods (adsorption experiments)

Two heavy metal stock solutions of 50 ppm were prepared in line with the European Pharmacopeia. Calibration curves of 25, 100, 250, 500, and 1000 ppm stock solutions of the heavy metals were prepared by diluting the solutions with deionized water. Simultaneously, solutions of 50, 75, 100, 150, 250, 350, 500, 750, 850, and 1000 ppm required for performing the experiments were prepared following the same procedures. The uptake of Pb and Cd heavy metals was tested using a batch process. Tests were performed in 250 mL Erlenmeyer flasks that were stirred at 400 rpm. After filtering the heavy metal solutions from the CKD powder, the heavy metals' final concentrations were detected using microwave plasma-atomic emission spectroscopy (MP-AES), Agilent 4200/4210 MP-AES, USA.

The HMs removal capacity at equilibrium *Q*_e_ (in mg g^−1^) and the HMs removal percentage from solution (*R*%) were calculated using eqn (1) and (2).^7^1
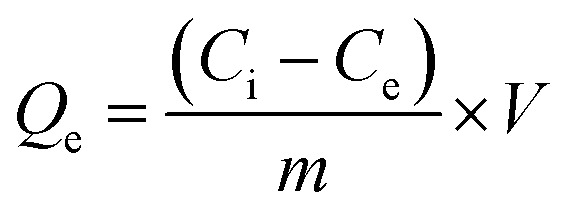
2
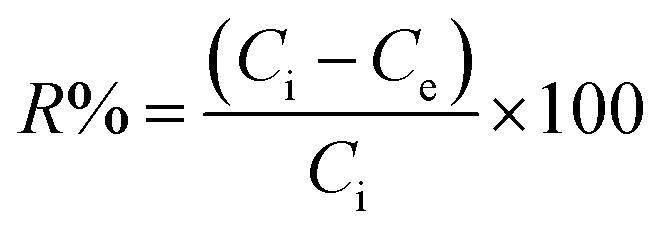
Here, *C*_i_, *C*_e_, *m*, and *V* are the starting metal concentration (mg L^−1^), HMs concentration during equilibrium (mg L^−1^), the beginning CKD mass (g), and the solution volume in (L) that was set to 0.1 L for all the tests, serially.

#### 2.2.1 Contact time effect and investigating the kinetic modeling

The magnitude of adsorption was quantified *vs.* time to comprehend the kinetics of Pb and Cd adsorption. The experiments were executed by blending 0.10 g of CKD with 1000 ppm Pb and Cd ion solutions at ambient temperature for time intervals of 15, 20, 30, 40, 50, 60, 70, 80, 90, and 100 minutes at a constant pH of 8.

For kinetic modeling, pseudo-first-order, pseudo-second-order, and intraparticle diffusion models (in both linear and nonlinear formats) were employed to identify the adsorption mechanism of the HMs on CKD powder by applying the detailed equations that are referred to in ref. 19 and 20.

#### 2.2.2 The effect of pH

In general, an adsorbent's surface charge is affected by the solution pH, which should be discussed to determine the best conditions for HMs removal. This can be achieved by conducting tests with initial pH levels of 2, 4, 6, 8, 10, and 12 for solutions containing 0.1 M HCl and NaOH. The experiments were conducted by blending 0.10 g of CKD with 1000 ppm Pb and Cd suspensions for a contact time of 90 min at atmospheric temperature.

#### 2.2.3 The impact of modifying the heavy metals' initial concentrations and investigating the isotherm models

Different initial heavy metal concentrations of both Pb and Cd, specifically 50, 75, 100, 150, 250, 350, 500, 750, 850, and 1000 ppm, were examined with 0.10 g of CKD at ambient temperature, pH 8, and for 90 min of contact time. Langmuir and Freundlich isotherms were then applied using the obtained data to understand the nature of the interaction between the heavy metals and the adsorbent surface. The Langmuir model is founded on the idea that the soluble heavy metal is adsorbed as a monolayer at certain homogeneous points over the adsorbent surface, where all the sites are alike and energetically equal.^21^ The Langmuir linear style is mathematically expressed as:3
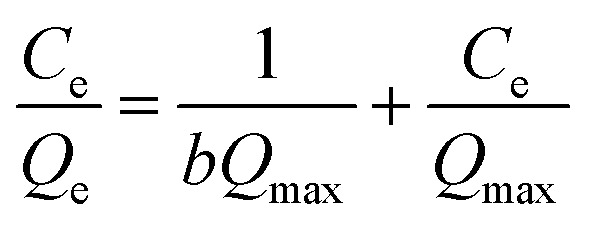
where *b* and *Q*_max_ are the Langmuir constant of the adsorption process (L mg^−1^) and the highest capacity of heavy metal at entire monolayer coverage per mass unit (mg g^−1^), respectively. The values of *b* and*Q*_max_ can be identified from the intercept and slope of a plot of 
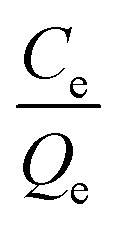
*versus C*_e_.

The Langmuir separation equilibrium constant (*R*_L_), which is the prime factor of the model, can be derived from eqn (4).4
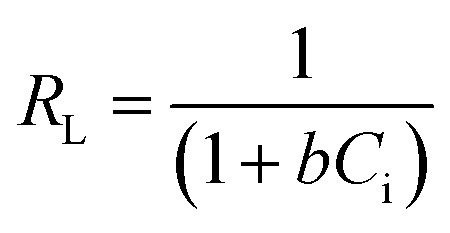
Here, *C*_i_ denotes the starting concentration of metal in the solution. The values of *R*_L_ can be utilized to identify the adsorption category. An *R*_L_ value of 0 denotes irreversible adsorption, and that the adsorbate cannot be eliminated. A value in the range 0–1 signifies that the adsorption is favorable. A value higher than 1 denotes that the adsorption is conditionally unfavorable. Lastly, a value of *R*_L_ = 1 signifies linear adsorption activity.^20^

The nonlinear form of the Langmuir isotherm is expressed by eqn (5).5
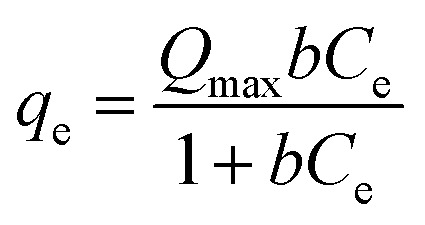


The Freundlich adsorption model is employed to illustrate the non-ideal heterogeneous multilayer adsorption. It presumes that adsorption happens on heterogeneous surfaces and incorporates multiple dissimilar locales with different adsorption energies.^22^ The model is linearly formulated as shown in equation (6).

6 ln *Q*_e_ = ln *K*_f_ + 1/*n* ln *C*_e_

where, *K*_f_ and *n* are the Freundlich constants that denote the adsorption load (mg g^−1^), and the adsorption extremity, respectively, which can be calculated from the slope and intercept of a plot of ln *Q*_e_*vs.* ln *C*_e_.

The nonlinear mode of the Freundlich isotherm^23^ is denoted as follows.

7 *Q*_e_ = *K*_f_*C*_e_^1/*n*^

#### 2.2.4 Influence of cement dust mass

Various amounts of CKD, namely, 0.10, 0.20, 0.30, 0.40, and 0.50 g, were exposed to 1000 ppm solutions of Pb and Cd ions at ambient temperature, pH 8, and for 90 min of contact time to study the influence of CKD mass on adsorption.

#### 2.2.5 Effect of altering the temperature and study of the thermodynamic parameters

The effect of working temperature on adsorption was studied by blending 0.2 g of CKD with 1000 ppm of Pb and Cd suspensions at 25, 35, 45, and 55 °C (298, 308, 318, 328K), with a constant pH of 8 and a contact time of90 min. Based on this, thermodynamic parameters (Δ*G*°, Δ*H*°, and Δ*S*°) were mathematically determined by applying the equations in ref. 22.

### 2.3 Characterization

#### 2.3.1 CKD chemical composition

The form of the neat CKD powder was determined using an Axios Max Panalytical wavelength dispersive XRF spectrometer, UK.

#### 2.3.2 Determination of the point of zero charge

The point of zero charge (PZC) of the CKD, applying the drift approach, was determined by adding 50 mL of a 0.1 M KNO_3_ solution to a set of 100 mL conical flasks. A pH range of 2–12 was obtained by adding drops of HNO_3_ and NaOH (0.1 mol L^−1^) to the solution. A CKD dose of 2 g L^−1^ was then added to the flasks and stirred for 24 h at room temperature, and the final pH values were measured using a Jenway 3510 pH meter (UK). The initial pH *versus* ΔpH (pH_initial_ − pH_final_) was plotted to determine the PZC of the CKD, as shown in ref. 18.

#### 2.3.3 Fourier-transform infrared spectra

The FTIR spectra of CKD rich with heavy metals were obtained using an FTIR spectrometer (InfraRed Bruker Tensor 37, Germany). The powder for each sample was mixed with the KBr carrier and pressed into a disk shape. The examination was executed in the range of 4000–400 cm^−1^, applying a resolution of ±2 cm^−1^ with 0.01 cm^−1^/2000 cm^−1^ accuracy at room temperature.

#### 2.3.4 X-ray diffraction study

The crystallography of the neat CKD and the CKD after adsorption, accompanied by the contained phases, was identified using a Bruker MeasSrv (D2-208219)/D2-2082019 diffractometer that runs at 30 kV, 10 mA, and that has a Cu Kα radiation tube (*λ* = 1.5406 Å), USA. The examination was carried out in the 2*θ* detection range of 0–100° with a high-resolution step of 0.01°.

#### 2.3.5 Weight loss determination

Thermogravimetric (TGA) analysis curves of the examined samples were investigated using a Universal TA instrument V4.5A, SDT Q600 V20.5 Build 15 device, USA. Around 5–20 mg of the samples (in powder form) was put in a platinum pan and heated at 10 °C min^−1^ from room temperature to 850 °C in a nitrogen atmosphere with a flow rate of 10 mL min^−1^. The accuracy of the device was ±1 °C.

#### 2.3.6 Scanning electron microscopy

The morphological details of the CKD before and after Pb and Cd metal adsorption were determined by using a JEOL-JSM-IT200 apparatus. Before the examination, the CKD particles were first coated with a gold thin layer using a JFC-1100E JEOL ion sputtering apparatus.

### 2.4 Models and fittings

The two Langmuir models and the Freundlich model were employed (linearly and nonlinearly) to determine the relation between the load of adsorbed substance and its concentration in the suspension at a fixed temperature once equilibrium is reached. These models were also applied to establish the adsorption mode (the interaction) at the adsorbent-sorbate border.^48^ The Langmuir model assumes that the adsorption takes the form of a monolayer coverage of the adsorbate over a homogeneous adsorbent shell at homogeneous locations with equivalent energies. Conversely, the Freundlich hypothesis presumes that the surface of the adsorbent has available adsorption points with numerous powerful attachment energies at which the adsorption of the metal ions can occur in multi-layers, and that the quantity of these adsorbed materials is directly related to their concentration.

## 3 Results and discussion

### 3.1 Characterization of neat and used CKD (PZC, FTIR, XRD, TGA, and SEM)

**Fig. 1:**
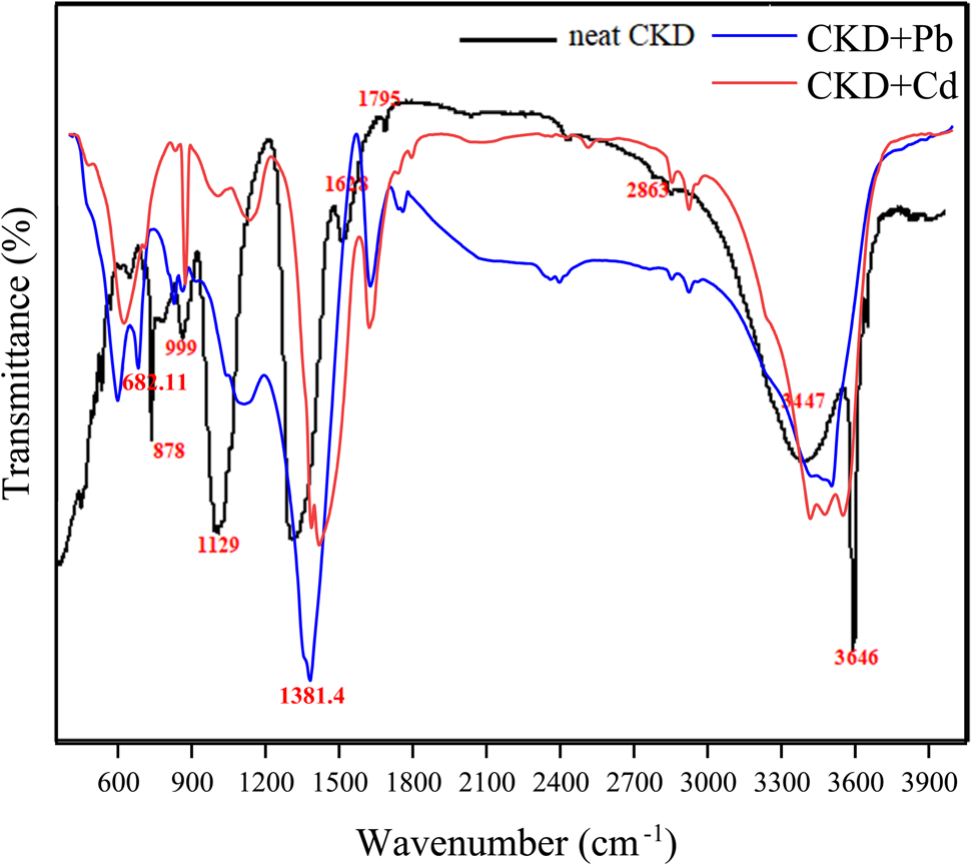
FTIR spectra of the neat CKD and CKD loaded with Pb and Cd metals.

**Fig. 2:**
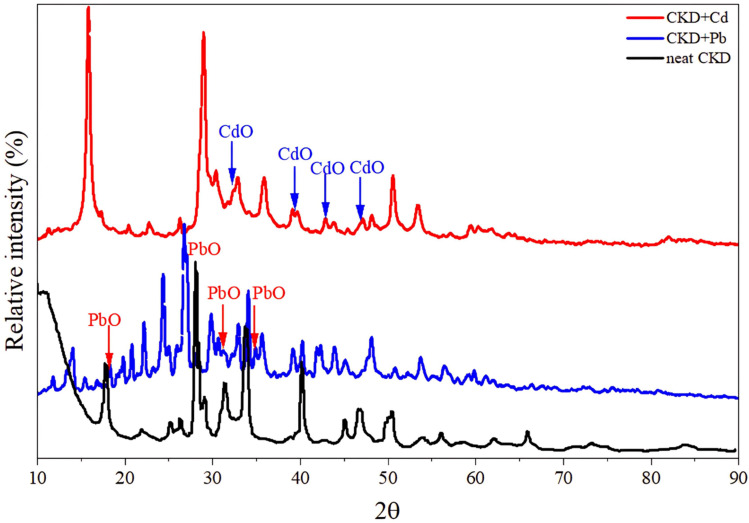
X-ray diffraction spectra of CKD and CKD loaded with Pb and Cd metals.

**Fig. 3:**
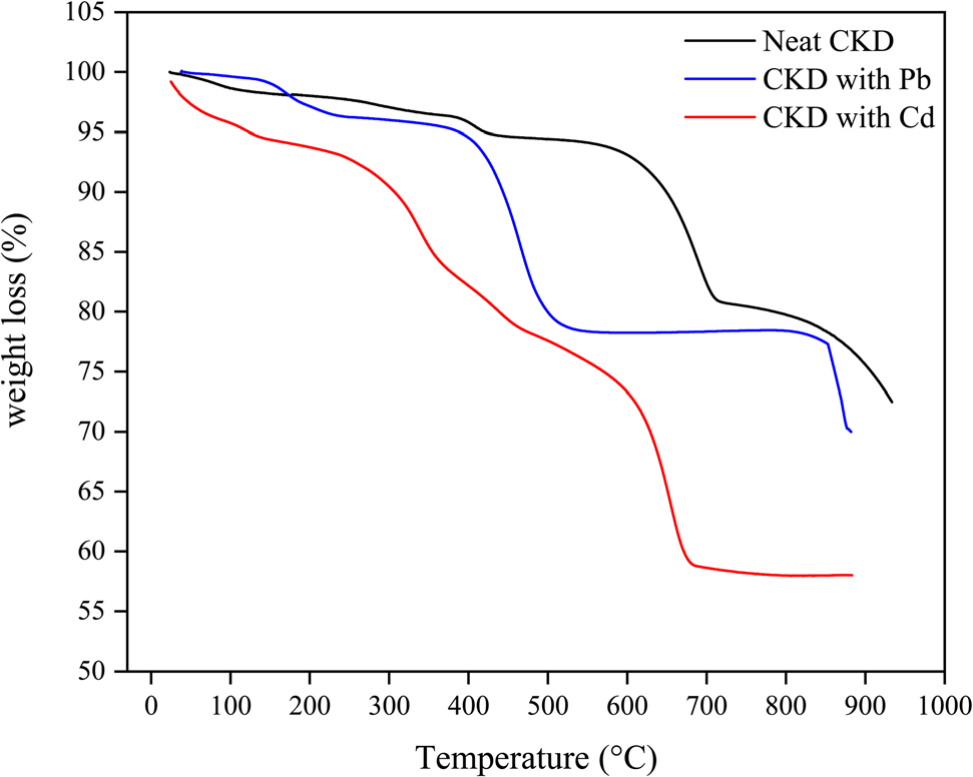
TGA thermograms of CKD and CKD loaded with Pb and Cd metals.

**Fig. 4:**
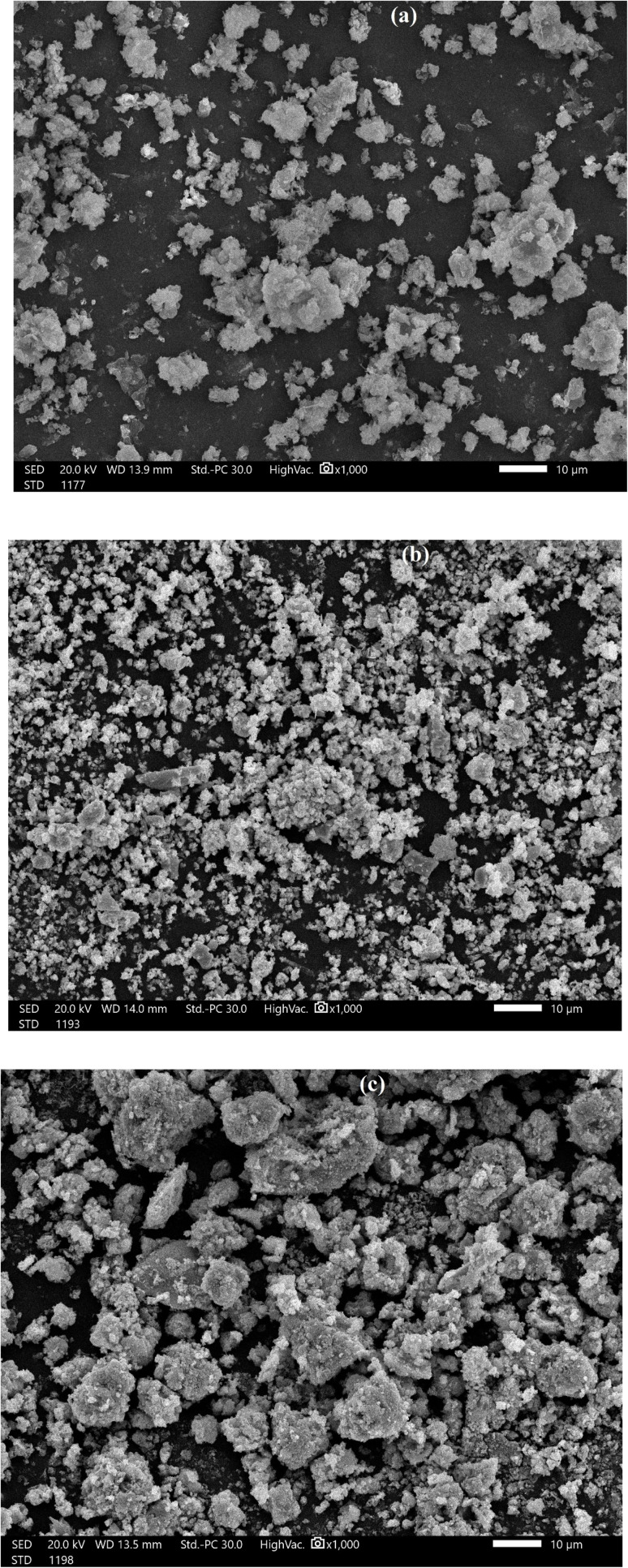
SEM images of (a) neat CKD, (b) CKD after Pb adsorption, and (c) after Cd adsorption.

The CKD powder chemical content is indicated in Table 1, in which almost 50% of the CKD composition is active lime. The CKDs' PZC was determined from the intersection of the initial pH with ΔpH and was found to be 8.6. At this value, the CKD surface exists in a neutral state and is not affected by the presence of either anionic or cationic functional groups. The 8.6 PZC value was determined in a previous work,^18^ and it is consistent with previous studies of CKD.^7^ The effect of the PZC on the uptake of HMs will be discussed later in the subsequent sections Fig. 1 reveals the FTIR spectra of CKD after adsorption of the Pb and Cd metals. The analysis of the FTIR spectrum of the virgin CKD was discussed in an earlier work.^18^ Concerning the FTIR spectrum of CKD loaded with Pb, the characteristic peaks at both 682.11 and 1381.4 cm^−1^ are the peaks related to the alpha and beta forms of lead oxide.^24^ The two very small shoulders observed at 712 and 828.4 cm^−1^ in the spectrum of CKD loaded with Cd are ascribed to the Cd–O bond.^25,26^ The transmittance peaks of the pure CKD were all shifted from their original positions to new positions, as detailed in Table 2. The appearance of peaks related to lead and cadmium oxides, and also the observed shifts of the CKD peaks and the changes in their intensities, confirm the interaction and the attachment between CKD and both the Pb and Cd metals. The XRD crystallography patterns of the crude CKD before and after the adsorption of the Pb and Cd metals are shown in Fig. 2. The pristine CKD was analyzed in an earlier work.^18^ The results of the XRD study match those of the XRF study (Table 1). After the adsorption of the HMs, the XRD spectra of the Pb- and Cd-sorbed CKD showed the presence of new peaks at 18°, 28.5°, 31.8°, and 35.7° that correspond to PbO and peaks at ≈32°, 39°, 42.7°, and 47.3° that are related to CdO, as confirmed by a literature survey.^24,26–29^ As both Pb and Cd exist in their usual oxidation states and the CKD provides an oxygen-rich environment, this facilitates the interaction of the metals with oxygen, allowing the formation of the PbO and CdO that appear in the XRD spectra.^30^ The appearance of these new peaks related to PbO and CdO confirms the adsorption of the Pb and Cd metals by the CKD, and, owing to the appearance of metals in the form of oxides, that this interaction is chemical by nature, *i.e.*, substitution/ion precipitation. The results obtained from the TGA analysis of the pure CKD and CKD after the adsorption of both the Pb and Cd metals are shown in Fig. 3. The CKD thermogram reveals different weight loss stages starting in the range 340–455 °C. The thermal decomposition at this stage is ascribed to the breakdown of Ca(OH)_2_ in CKD into minor CaO and H_2_O species (dehydration).^31,32^ The subsequent sharp thermal weight loss stage, about 14%, in the range 460–770 °C, is possibly related to the dehydration of the amorphous C–S–H phase at which the largest weight reduction occurs and the growth of the wollastonite (CaSiO_3_) crystalline phase.^33^ In the case of CKD loaded with Pb, the first small weight loss, approximately 4%, observed at about 170 °C is ascribable to the evaporation of moisture. The second weight loss step, about 18%, which has a peak centered at ≈450 °C, is due to the thermal decay of the existing inorganic PbO_2_ to PbO at different overlapping stages and includes a series of decomposition transformations as follows: PbO_2_ to PbO_1.89_ at ≈360 °C; PbO_1.89_ to PbO_1.59_ at ≈405 °C; PbO_1.59_ to PbO_1.43_ at ≈409 °C; PbO_1.43_ to PbO_1.29_ at ≈460 °C; and finally the decomposition of PbO_1.29_ to PbO at about 512 °C.^34,35^ The thermogram of CKD loaded with Cd shows the first small observed weight loss peak at about 150 °C, which is due to the evaporation of the coordinated moisture.^36^ The second and third weight reduction stages, occurring at ≈250 and 670 °C and accounting for about 37% of the total weight loss, are mainly attributed to the presence of cadmium oxide. This typically matches the prior literature.^37–39^ All the outputs of this section, the formation and the presence of both lead and cadmium oxides, can be linked with the FTIR and XRD results (Fig. 1 and 2) and confirm that they are related to the chemical interaction between the heavy metal species and CKD. The SEM electron photomicrographs of the CKD before and after loading with HMs are presented in Fig. 4. Fig. 4(a) shows the SEM image of the neat CKD and reveals the presence of gaps and apertures in its structure that are flaky or strip-like, particularly when the image is taken at elevated amplification power. This matches what was revealed by earlier literature that reports the characterization of CKD.^3,30^ The photomicrographs indicate a highly fine porous and irregular structure with a high surface area^40,41^ that provides a high number of available reacting adsorption sites for heavy metals, facilitating their removal process.^42^ By examining the morphology of the CKD before and after adsorption, it is found that the texture of the CKD surface is altered after the adsorption of both Pb (Fig. 4(b)) and Cd (Fig. 4(c)). The HMs appear as unique growths on the exterior (strips) of the CKD, which is covered with new microcrystalline white precipitates. The intermolecular spaces of the CKD surface are also filled by these molecules (*i.e.*, visible evidence of reduction in the pores was obtained) in monolayers and multi-layers to create surfaces with dense morphologies. This evidence demonstrates the successful binding of the Pb/Cd HMs (adsorbate) on the surface of the CKD (adsorbent) *via* various mechanisms.^30^

**Table 1:** Chemical composition of cement dust as obtained using X-ray fluorescence spectroscopy

Oxide	wt%
SiO_2_	13.48
Al_2_O_3_	2.99
Fe_2_O_3_	3.60
CaO	49.24
MgO	3.43
SO_3_	4.42
Na_2_O	2.80
K_2_O	6.34
Cl	8.58
LOI	7.58

**Table 2:** The shifts of the CKD transmittance peaks after the adsorption of Pb and Cd metals

Peak attribution	Original peak position in the CKD spectrum (cm^−1^)	The peak position after Pb sorption by CKD (cm^−1^)	The peak position after Cd sorption by CKD (cm^−1^)
Bending vibrations of the Si–O bonds in both the C_2_S and C_3_S phases	878	863.15	874.01
Bending vibrations of the Si–O bonds in both the C_2_S and C_3_S phases	842	828.40	833.08
Bending vibrations of the Si–O bonds in both the C_2_S and C_3_S phases	1129	1113.73	1136.58
Si–O–Si bond	999	—	1006.57
Si–O–Si asymmetric vibration overlapped with the aromatic C=C peak	1628	1626.86	1622.45
O–H (hydroxyl)	1795	1791.38	1794.54
C–H stretching in alkanes	2853	2853.40	2854.59
O–H stretching vibrations of the adsorbed water on the cement kiln dust phases	3474	3468	3476.75
Asymmetric stretching of carbonate CO_3_^2−^ molecules resulting from the reaction of calcium hydroxide with the air atmosphere	1417	—	1419.36

### 3.2 The effect of changing various parameters on the uptake of lead and cadmium by the cement kiln dust

#### 3.2.1 Influence of altering cement kiln dust mass on metal removal

**Fig. 5:**
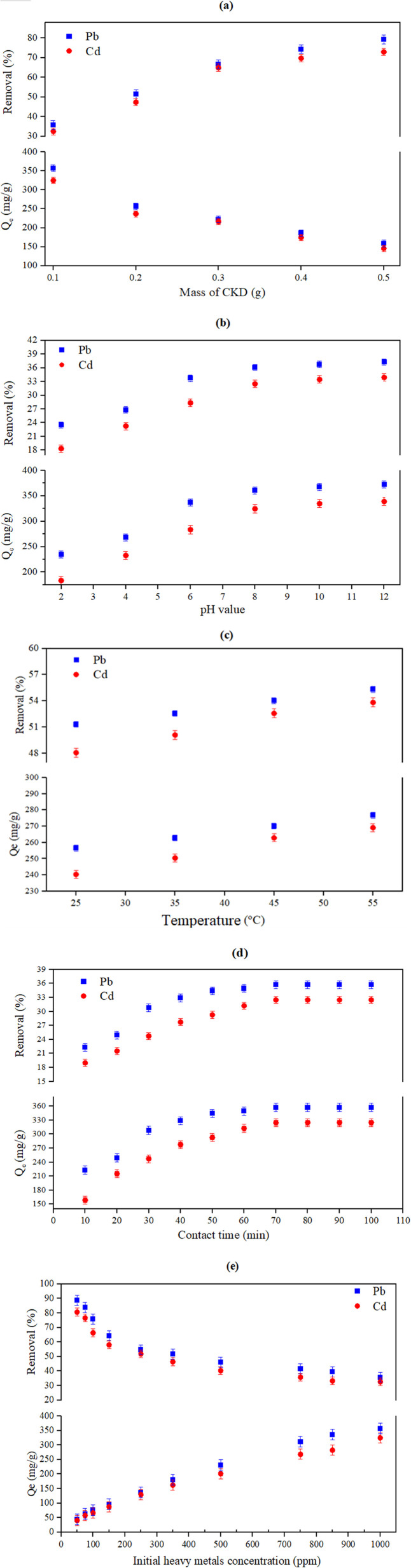
The effect of varying (a) the CKD mass (from 0.1 to 0.5 g with 1000 ppm metal concentrations), (b) pH value (with 0.1 g of CKD and 1000 ppm metals concentration), (c) temperature (with 0.2 g of CKD and 1000 ppm metals concentration), (d) contact time (with 0.1 g of CKD and 1000 ppm metals concentration), and (e) the initial heavy metal concentrations (varied from 50 to 1000 ppm with 0.1 g of CKD) on both the removal (%) and the amount (in mg g^−1^) of equilibrium adsorption (*Q*_e_) of the Pb and Cd metals.

The effect of changing the initial amount of CKD on the removal of Pb and Cd metals, along with their bulk of equilibrium adsorption (in mg g^−1^) was investigated, and the results are shown in Fig. 5(a). From the figure, one can notice that the uptake efficiency of the CKD for both Pb and Cd dramatically increases from 35.7% to 79.2% for Pb and from 32.4% to 72.9% for Cd when the CKD mass used is increased from 0.1 to 0.5 g. This increase in removal is due to the ability of CKD, which is characterized by small granules and, thus, small pore volumes, to provide a large surface area per unit and, therefore, more adsorption locales (vacant sites). Conversely, increasing the CKD mass may lead to more linkages between the active sites on its surface, which could explain the observed diminution in the equilibrium adsorption of the Pb/Cd metals when the CKD mass used is increased.^43^ To better understand the effect of CKD on the adsorption of the metals, the adsorption of both Pb and Cd metals at pH 12 without the presence of CKD was performed. The obtained data revealed that significant removal of both Pb and Cd occurred at pH 12 due to Pb(OH)_2_ and Cd(OH)_2_ precipitation. These findings support the fact that a considerable fraction of metal removal at alkaline pH can be attributed to precipitation rather than the adsorption mechanism.

#### 3.2.2 The effect of changing the pH of the solution on metal removal

The pH of the media is a factor that modifies the adsorption of the metal ions by influencing both the surface charges and the CKD functional groups, along with the ionization degree of the metal ions. The effect on the metal ion removal caused by shifting the pH values from 2 to 12 is displayed in Fig. 5(b). The figure indicates that the adsorption of Pb and Cd ions significantly rises from 234.9 to 372.7 mg g^−1^ for lead and from 183.3 to 339 mg g^−1^ for cadmium when the pH is increased. The highest uptake of the metal ions was attained in the 6–8 pH range, followed by a very slight increase (near steady state) in the adsorption at higher pH levels. The adsorption mechanism of both the Pb and Cd ions on CKD as a function of pH is mainly directed by the interplay of the modification of the surface charge on the CKD and the metal ions, and by possible precipitation effects. In detail, at lower pH, the adsorbent surface is predominantly positively charged due to the protonation of functional groups on the CKD, which causes electrostatic repulsion with the cationic metal ions (Pb^2+^, Cd^2+^), resulting in a lower adsorption capacity. Moreover, a high concentration of H^+^ ions competes with metal ions for the binding sites, reducing the sorption efficiency. In the pH range of 6–8, deprotonation of the CKD surface functional groups occurs. This increases the negative surface charge, enhancing the electrostatic attraction between the CKD surface and the metal ions and thus maximizing adsorption capacity. The metal ions remain primarily in their free soluble ionic forms, favoring the uptake process. Beyond pH 8, the sorption uptake reaches a plateau, likely due to the probable onset of metal hydroxide precipitation in the form of insoluble micro-precipitates. This precipitation reduces the availability of free ions for more adsorption and may also block active adsorption sites, thus obstructing further uptake despite the increasing pH. This pH-dependent sorption behavior reflects an intricate balance between the electrostatic interactions, surface complexation, the ionization states of the CKD functional groups, and metal speciation equilibria, consistent with the findings of Waly *et al.*, Cheng *et al.*, El Zayat and El Zayat *et al.*, who observed similar trends in Pb and Cd removal using CKD and hydrated Portland cement.^1,3,7,16^

#### 3.2.3 Effect of temperature on heavy metal uptake

The effect of increasing the mixture temperature from 25 to 55 °C on the adsorption of the Pb/Cd metals and their*Q*_e_ values is shown in Fig. 5(c). The results demonstrate that the HMs uptake shifts upward from 256.5 to 276.6 mg g^−1^ for lead and from 240.31 to 269.01 mg g^−1^ for cadmium with an increase in the temperature. This is conceivably due to the increase in the kinetic mobility of the adsorbate particles at elevated temperatures, which increases their mobility and accelerates their diffusion rate through the solution in the vicinity of the outside layer and the inward voids of the CKD, thus increasing the chance of interaction between the HMs and CKD.^46^ More evidence for the rise in the HMs uptake with temperature is the thermodynamic favorability of the adsorption process due to its endothermic nature, which is confirmed by the thermodynamic parameters (positive Δ*H*, and entropy change (Δ*S*°) and favorable Δ*G*°) as will be discussed later.

#### 3.2.4 Kinetics study

**Fig. 6:**
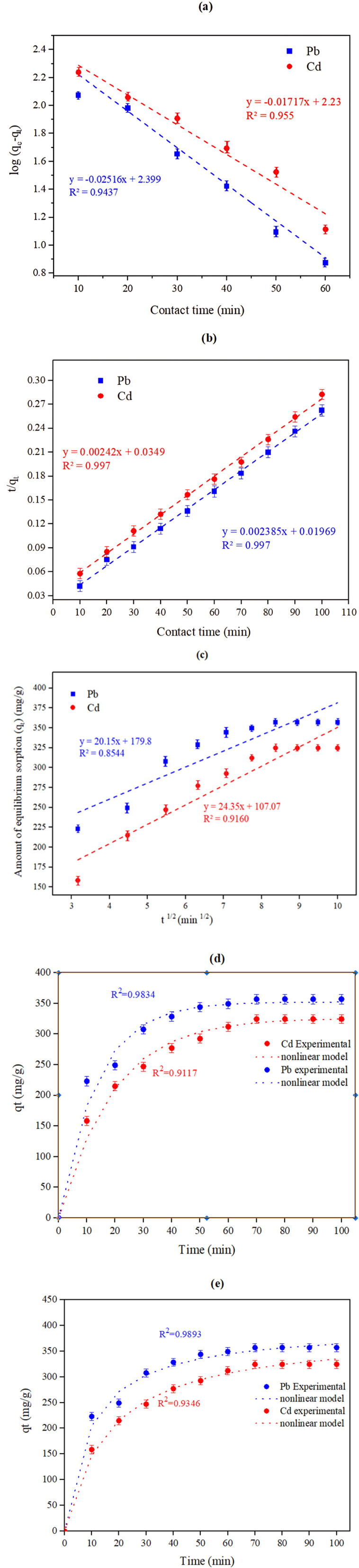
Linear (a) Pseudo-first-order, (b) linear pseudo-second-order, (c) linear intra-particle-diffusion, (d) non-linear pseudo-first-order, and (e) non-linear pseudo-second-order kinetic model plots for the adsorption process of Pb and Cd heavy metals on the surface of the CKD particles.

The contact time is identified as the necessary time to equalize the adsorption/desorption rates or the time required by the material to attain a saturation equilibrium state.^44^ The effect of changing the contact time from 10 to 100 minutes on the adsorption process was studied and is shown in Fig. 5(d). The graphical illustration of contact time exposure reveals an effective increment in the metal ion removal% in the first 70 minutes from 223.01 to 357.1 mg g^−1^ for lead and from 158.4 to 324.6 mg g^−1^ for cadmium. After that, a peak in the removal at a contact time of 80 minutes is achieved. After this time, the metal adsorption process was not governed by time (*i.e.*, it is time-independent) and reaches a plateau. The effective adsorption in the first 70 minutes is due to the abundance of accessible vacant adsorption sites before equilibrium is reached. Following this, these sorption points are saturated with metal ions, decreasing the chances for further adsorption. Finally, dynamic equilibrium is achieved at the highest value (70 minutes), and at that moment, the rates of both adsorption and desorption become equal.^45^ This illustrates that the adsorption is contact time-dependent. The steady-state adsorption time achieved here was reached after a time nearly identical to the equilibrium adsorption times of other heavy metals, like Zn, Al, Co, and Cd, when adsorbed by CKD.^1^ Consequently, the linear pseudo-first-order, pseudo-second-order, as well as intraparticle diffusion kinetic models were employed to establish the mechanism of adsorption of the HMs on the CKD, as shown in Fig. 6(a–c). The kinetic variables that these models utilize are listed in Table 3. The data presented in Fig. 6 and Table 3 exhibit good agreement between the kinetic results and a pseudo-second-order model, demonstrating that this model fits the adsorption process at all the studied time points better than the other applied models. The adsorption capacity (*q*_e2_) for the linear form of the model is 392.7 (mg g^−1^) for Pb(ii) and 378.94 (mg g^−1^) for Cd(ii), as tabulated in Table 3. These values agree with the experimental adsorption capacities for lead and cadmium, which are 357.1 (mg g^−1^) and 324.6 (mg g^−1^), respectively. The regression coefficient (*R*^2^) of the model is 0.997, close to unity for both the adsorbed metals. These findings confirm that adsorption of the metals on the CKD surface is a chemical process that involves electron sharing between the adsorbate ions and the adsorbent pores, and that chemisorption is the dominant uptake mechanism for the existing heavy metals.^15,40^ Application of the nonlinear models to the kinetic data resulted in a satisfying fit and confirmed the applied linear models' results, as outlined in Fig. 6(d, e) and Table 3.

**Table 3:** Kinetic parameters for Pb and Cd adsorption on CKD obtained using various linear kinetic models (pseudo-first-order, pseudo-second-order, and the intra-particle diffusion model)

Kinetic model	Parameter	The value obtained for each metal	
		Pb	Cd
Pseudo-first-order	*k*_1_ (min^−1^)	0.0579	0.0395
	*Q*_e_ (mg g^−1^)	11.54	10.80
	*R* ^2^	0.943	0.955
Pseudo-second-order	*k*_2_ (g mg^−1^ min^−1^)	0.00028	0.00016
	*Q*_e_ (mg g^−1^)	392.7	378.93
	*R* ^2^	0.997	0.997
Intra-particle diffusion	*k_*i*_* (mg g^−1^ min^1/2^)	1.40	1.39
	*C* (mg g^−1^)	44.08	43.62
	*R* ^2^	0.854	0.916

#### 3.2.5 The effect of the heavy metal concentration on metal removal and discussion of the adsorption isotherm models

**Fig. 7:**
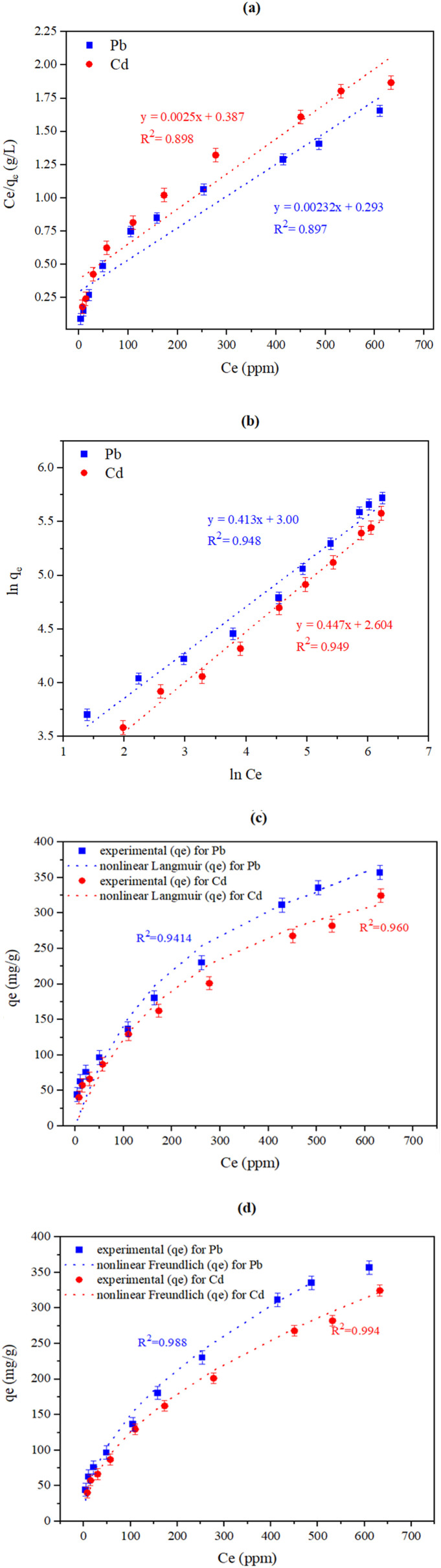
Linear (a) Langmuir and (b) Freundlich isotherm parameters and the nonlinear fittings of the (c) Langmuir and (d) Freundlich models for the adsorption of Pb and Cd on the CKD surface.

The effect that varying the initial concentrations of HMs from 50 to 1000 ppm has on the adsorption of both Pb and Cd is shown in Fig. 5(e). The figure indicates a decreasing tendency of removal from 88.6% to 35.7% for Pb and from 80.5% to 32.4% for Cd, and the inverse relationship between the removal steps and the density of the adsorbate. In these circumstances, the Pb(ii) and Cd(ii) adsorption capacities of CKD increased from 44.3 to 357.1 mg g^−1^ and from 40.3 to 324.6 mg g^−1^, respectively. The high metal adsorption (removal) at low HMs concentrations may be because the roughness of the CKD results in removal by chemisorption at low concentrations.^47^ The subsequent decrease in the removal of the metals at higher initial concentrations is due to the competition of the adsorbate atoms for the active sites of the adsorbent, which are increasingly filled as the metal concentrations increase from 50 to 1000 ppm. Another probable cause for the dwindling efficacy of the removal process is the reduction in the mass movement between the adsorbent and the solution at high metal concentrations.^7^ The behavior of the heavy metal adsorption in the current research is in complete agreement with previous research that has used CKD for the elimination of heavy metals.^16^Fig. 7(a, b) and Table 4 show the adsorption isotherm parameters for both metals, obtained using the Langmuir and the Freundlich models. Initially, it was observed that the separation factor (*R*_L_) values are 0.325 and 0.363, which are located between 0 and 1, suggesting that the adsorption process for both metals on CKD is favourable and proceeds efficiently under laboratory conditions. In addition, the values of the 1/*n* parameter of the Freundlich model are between 0.1 and 1, as indicated in Table 3, which also confirms that the removal process is favorable.^48^ Both these factors (*i.e.*, *R*_L_ and 1/*n*) will confirm and agree with the conclusions presented later in Section 3.3 (studying the thermodynamics of the adsorption process). Second, the validity of both the Langmuir and the Freundlich models in the current study is supported by the excellent fit observed for both the linear and nonlinear regressions in Table 3. This simultaneous validity can be rationalized by the fact that CKD possesses features of both homogeneous monolayers and heterogeneous multilayer surfaces, as evidenced by the quantitative parameters. The Langmuir model agrees well with the experimental data, due to the notable fraction of energetically equivalent sorption sites (monolayer, homogeneous) on the CKD surface as indicated by the *R*^2^ values (0.897–0.898 linear, 0.9414–0.960 nonlinear for both Pb and Cd, respectively). In parallel, the Freundlich model presents an excellent fit (linear *R*^2^ up to 0.948 for Pb and 0.949 for Cd and nonlinear *R*^2^ up to 0.988 for Pb and 0.994 for Cd), indicating that multilayer sorption and a variety of site energies also contribute significantly to the adsorption process and that this model accounts for the largest share of the adsorption process.^49^ Thus, while both models are valid, the Freundlich isotherm approach appears to better reflect the multi-energetic and heterogeneous nature of the CKD surface (rather than the Langmuir approach) under the experimental conditions due to higher *R*^2^ values of the model, as shown in Table 4. The heterogeneity of the CKD surface can also be confirmed from the *n* values of the Freundlich model (Table 4), which are >1, while the 1/*n* values of the model lie in the range 0.413–0.447, demonstrating the high affinity of Pb and Cd for the CKD surface and the occurrence of chemisorption.^48^ On the basis of the Langmuir and Freundlich isotherms, the interactions between the CKD and the Pb and Cd metal ions are a combination of physical and chemical adsorption on the CKD surface.^1,3,7,44^ Third, it was observed that the amount of adsorbed metal per one gram of CKD, *i.e.*, the adsorption capacities, were 403.7 and 362.5 mg g^−1^ for Pb and Cd, respectively. Conversely, the nonlinear Langmuir and the Freundlich isotherm models were employed for both metals to demonstrate the close match to the experimental data and to avert the attendant errors and biases that can possibly originate from linearization. The linear and nonlinear constants were computed and are compared in Table 4. The fittings and the deviations from the experimental data utilizing both models are illustrated in Fig. 7(c and d). The nonlinear *R*^2^ values for the two models support the above-mentioned linear findings, with a preference for the Freundlich model in describing the adsorption process.

**Table 4:** Linear and non-linear parameters for the Langmuir and Freundlich adsorption isotherms for Pb and Cd ions on CKD surface

Metal	Langmuir model						Freundlich model							
	*b* (L mg^−1^)	*Q*_e_ (mg g^−1^)	*Q*_max_ (mg g^−1^)	*Q*_max_ (nonlinear)	*R* ^2^	*R* ^2^ (nonlinear fit)	*R* ^L^	*K*_f_ (mg g^−1^)	*K*_f_ (mg g^−1^) (nonlinear fit)	*n*	1/*n*	*n* (nonlinear fit)	*R* ^2^	*R* ^2^ (nonlinear fit)
Pb	0.00764	357.1	403.7	472.61	0.897	0.9414	0.3254	21.5	14.56	2.26	0.413	1.972	0.948	0.988
Cd	0.00625	324.6	362.5	423.06	0.898	0.960	0.3638	14.53	12.20	2.04	0.447	1.969	0.949	0.994

### 3.3 Studying the thermodynamics of the adsorption process

**Fig. 8:**
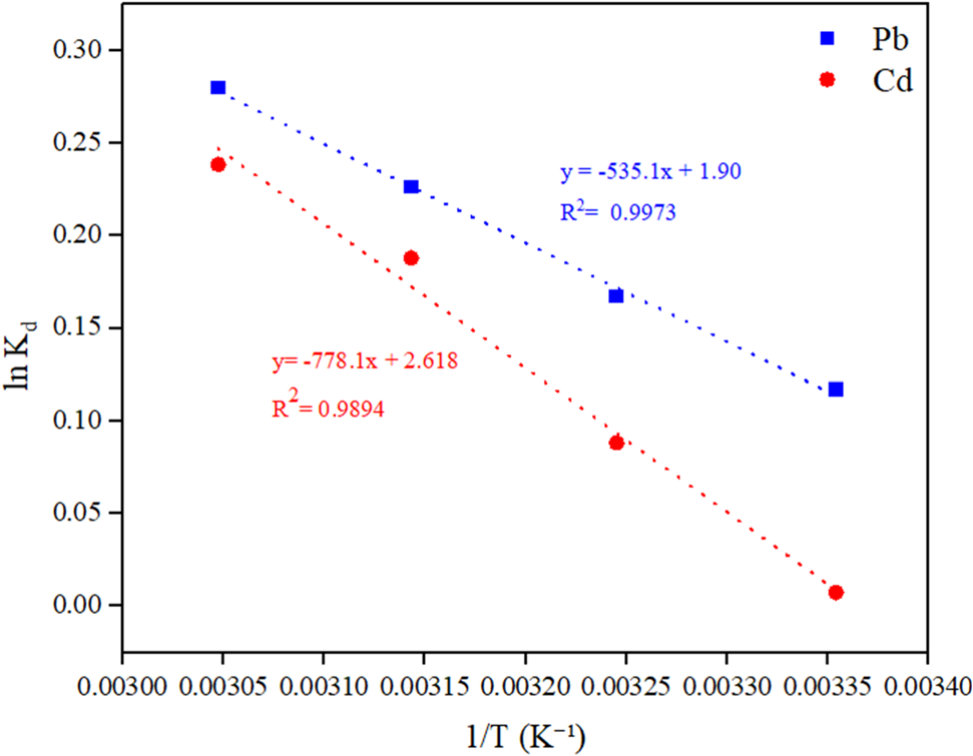
Van ‘t Hoff plot used to obtain the adsorption thermodynamic parameters of Pb(ii) and Cd(ii) on cement kiln dust (CKD): linear fits of ln *K*_d_*versus* 1/*T* (K^−1^) were used to obtain Δ*H*° from the slope −Δ*H*°/*R* and Δ*S*° from intercept Δ*S*°/*R*. The corresponding regression equations and *R*^2^ values are also shown.

The values of Gibbs free energy changes (Δ*G*°), enthalpy changes (Δ*H*°), and entropy changes (Δ*S*°), the standard thermodynamic parameters, were obtained through the classical van ‘t Hoff method (the intercept and slope of ln *K*_d_*vs.* 1/*T*) as plotted in Fig. 8. From the linear relation ln *K*_d_ = −Δ*H*°/(*RT*) + Δ*S*°/*R*, the slope provides the standard enthalpy change Δ*H*°, while the intercept gives the standard entropy change Δ*S*°, allowing calculation of Δ*G*° at each temperature *via* the relation Δ*G*° = Δ*H*° − *T*Δ*S*°, which can be checked with Δ*G*° = −*RT* ln *K*_d_. Table 5 lists the thermodynamic variables for the Pb/Cd metals. From Table 5, the endothermic nature of the current adsorption activity of the metals on the CKD can be verified owing to the positive value of Δ*H*. In addition, the negative (Δ*G*°) values at all the tested temperatures (298–328 K) signify that the adsorption trajectory is energetically favorable and unrestrained from the thermodynamic point of view under the experimental conditions. The positive Δ*S*° values denote a positive attraction of metal ions to the CKD, and that their mobility is not confined and that they grow irregularly at the liquid/solid frontier with increasing temperature and time of adsorption, which is consistent with the findings in Section 3.2.3 (effect of temperature on heavy metal uptake).^20^ These findings are in complete harmony with preceding studies that applied CKD in the removal of metal ions.^15^

**Table 5:** Thermodynamic parameters of Pb and Cd adsorption on the CKD particles

Metal	Δ*G*° (kJ mol^−1^)				Δ*H*° (kJ mol^−1^)	Δ*S*° (J mol^−1^ K^−1^)
	298 K	308 K	318 K	328 K		
Pb	−0.29	−0.43	−0.60	−0.76	4.46	0.0159
Cd	−0.018	−0.22	−0.50	−0.65	6.489	0.0218

### 3.4 Suggested removal mechanism

The XRF results showed that the major ingredients of the CKD are oxides. The metal cations of these oxides cause the alkalinity of the CKD to rise, creating opportunities for the formation of surface complexes between the CKD active sites and metal ions, causing the metal ions' capture/adsorption to increase *via* the adsorption interaction mechanism. The replacement of both Pb and Cd with either Ca^2+^ and/or Si^4+^ ions found in the cement functional groups (where both CaO and SiO_2_ represent two-thirds of the CKD ingredients and they behave as the adsorbent groups for HMs due to their high pH and reactivity)^50–52^ and the disappearance of the lead and cadmium oxide peaks in the FTIR and XRD spectra (Fig. 1 and 2) support the proposal that chemical ion exchange in the liquid phase makes up the largest share of the removal process, a process which involves both physical and chemical removal mechanisms.^3,40^ Furthermore, the high alkalinity of the CKD (≈10–12), where CaO is mainly responsible for this, plays a significant role in increasing the negative charges in solution, which in turn enhances the electrostatic interactions between the CKD and the positive ions of the heavy metals during the adsorption process.^15,47^

### 3.5 Real wastewater sample treatment (case study)

The aforementioned removal conditions were applied to a real wastewater sample obtained from inside a battery factory in the industrial area in Sadat City, Monufia Governorate, Egypt. The real industrial wastewater sample was agitated with 0.5 g of CKD at a 400 rpm stirring velocity for 80 min of contact time to assess the removal of HM traces from this real sample. Both the removal efficiency and the adsorption capacity were measured as well. Of course, the sample contained other HMs and pollutants, but our examination focused only on the studied metals (*i.e.*, Pb and Cd) to evaluate the applicability of using CKD in the purification of real wastewater in the industrial field. The initial concentrations of the Pb and Cd metals in the sample were 8.89 and 3.61 ppm, respectively. After applying the aforementioned working parameters and executing the adsorption process, the final concentrations of both metals in the real sample were detected using the MP-AES technique and were found to be 0.82 and 0.48 ppm with removal efficiencies of 90.77 and 86.70%, respectively. This affirms the suitability, selectivity, and utility of CKD as a low-cost adsorbent for heavy metals removal, especially when compared with other adsorbents from either organic sources, like activated carbon and chitosan,^53–55^ or from inorganic sources, like marble/granite composites,^56^ or even high-cost preparation adsorbents like carbon nanotubes (either single-walled or multi-walled).^57^ To completely address the environmental concerns of the treated water and the potential effects of CKD on the water being treated, a post-adsorption analysis was conducted on the purified water to evaluate the release of CKD's major constituents (such as Ca, Cl, …) into the treated water. After equilibrium was reached, the mixture (which contains CKD) was filtered to separate the CKD carrying the heavy metals. The filtrate was analyzed using MP-AES spectroscopy to quantify any leached elements. The reference purified wastewater, without CKD, was also analyzed to subtract possible background contributions. The results showed that the leaching of problematic elements, such as Al and Fe, was negligible. No statistically significant increase in either Si or Mg was noted in the analysis. Concentrations of leached elements, particularly Ca^2+^, Cl^−^, and K^+^ exhibited marginal increases after the sorption but remained below the maximum allowable regulatory limits for irrigation water quality according to related local standards and laws, revealing that CKD is stable in aqueous media and that its use as an adsorbent does not pose a secondary contamination risk under the applied conditions, which aligns with the findings of recent related studies in Egypt.^58^

## 4 Conclusion

In this study, CKD was utilized as an adsorbent for metals from wastewater (either simulated or real), and the removal process was characterized using FTIR, XRD, TGA, and SEM techniques. The outcomes showed that the removal % increased with increases in the mass of the CKD used, the pH of the wastewater, the removal contact times (up to a plateau at 80 min), and the adsorption temperature, while a decrease in removal efficiency was noted at higher initial concentrations of the heavy metals. The high regression coefficient obtained at all adsorption stages from fitting the kinetic data from all the studied times demonstrated that a pseudo-second-order model agreed with the experimental data better than the other employed models. The nonlinear forms of the applied kinetic models affirmed the aforementioned results. Studying the thermodynamic parameters proved the endothermic character of the adsorption process and that the process is energetically favorable and spontaneous. Applying both linear and nonlinear Langmuir and Freundlich isotherm models to the obtained experimental data showed that both models were good fits and that both models can be applied to explain the removal mechanism. The presence of PbO and CdO in both the FTIR and XRD spectra, and the suitability of the Langmuir and Freundlich models to the experimental data, were strong evidence for a mixed physical/chemical adsorption mechanism, with the largest share being attributed to chemical adsorption. Finally, CKD has proved its suitability as an adsorbent for purifying heavy metals from industrial wastewater (either simulated or real) in various ways. First, the repurposing of CKD, an industrial byproduct, contributes to waste valorization and minimizes the environmental burden related to its disposal. Second, the utilization of CKD in the effective removal of toxic heavy metals, such as Pb and Cd, from wastewater can significantly mitigate the risks of water pollution and associated health hazards, supporting safer water discharge and reuse in various fields. Third, the high uptake and favorable adsorption mechanisms possessed by CKD suggest that it could be a cost-effective and sustainable alternative for wastewater management, particularly in regions with CKD availability, which aligns with the Sustainable Development Goals (SDGs), especially those relating to health and the environment.

## Author contributions

E. S: investigation, conceptualization, writing – original draft. W. M: methodology, W. A. S: review and editing, A. M. E: review and editing, M. E. E: supervision. All the authors have read and approved the paper.

## Conflicts of interest

The authors declare no competing interests.

## Data Availability

No primary research results, software, or code have been included, and no new data were generated or analysed as part of this review.
